# Neurodevelopmental Outcomes in Term Infants with Neonatal Hyperbilirubinaemia in China: A Retrospective Cohort Study Using the Griffiths Developmental Scales

**DOI:** 10.3390/jcm15135225

**Published:** 2026-07-03

**Authors:** Yanan Ma, Na Wang, Xiangli Bian, Kun Zhang, Jiayi Chen, Sainan Fan, Jinping Zhang

**Affiliations:** Department of Pediatrics, Shanghai Sixth People’s Hospital, Shanghai Jiao Tong University School of Medicine, Shanghai 200233, China; myn199008@163.com (Y.M.); wangna980310@126.com (N.W.); bian_xiang_li@163.com (X.B.); zhangk0809@126.com (K.Z.); chenjiayi188@163.com (J.C.); 17621670655@163.com (S.F.)

**Keywords:** neonatal hyperbilirubinaemia, bilirubin-induced neurologic dysfunction, Griffiths Developmental Scales, neurodevelopment, term infants

## Abstract

**Background:** Neonatal hyperbilirubinaemia (NHB) affects approximately 60% of term infants and is a recognised cause of bilirubin-induced neurologic dysfunction (BIND); however, its subclinical neurodevelopmental sequelae have not been well characterised at the level of specific developmental domains. **Objectives:** We aimed to use the Griffiths Developmental Scales–Chinese Edition (GDS-C) to characterise the domain-specific neurodevelopmental profile of term infants with NHB and to identify clinical risk factors for adverse outcomes. **Methods:** We conducted a single-centre, retrospective cohort study of 123 term newborns delivered between September 2019 and August 2023 at a tertiary hospital in Shanghai, China; 77 had NHB and 46 were healthy controls. Neurodevelopmental outcomes were assessed using the GDS-C at a median age of 46.9 months (interquartile range [IQR], 36.4–59.7) by a single certified examiner who was blinded to bilirubin status. A developmental quotient (DQ) below 85 (>1 SD below the standardised mean of 100) or at or below the 10th percentile was classified as below cutoff; otherwise, performance was classified as within the normal range. To account for testing across six domains, a Bonferroni-adjusted significance threshold of *p* < 0.0083 was applied. **Results:** Compared to the control group, the jaundice group had higher proportions of below-cutoff performance in the Locomotor (26.0% vs. 10.9%; *p* = 0.045), Personal–Social (28.6% vs. 10.9%; *p* = 0.022), Eye and Hand Coordination (29.9% vs. 10.9%; *p* = 0.015) and Performance (35.1% vs. 15.2%; *p* = 0.018) domains. Among infants with severe hyperbilirubinaemia (total serum bilirubin (TSB) ≥ 342 μmol/L; *n* = 19), the proportions of below-cutoff performance in the Locomotor (47.4% vs. 19.0%; *p* = 0.032), Personal–Social (57.9% vs. 19.0%; *p* = 0.003) and Performance (57.9% vs. 27.6%; *p* = 0.034) domains exceeded those in the non-severe subgroup. Jaundice lasting ≥ 14 days was associated with poorer personal–social outcomes (*p* = 0.007). In multivariable logistic regression, both a peak TSB ≥ 342 μmol/L (adjusted odds ratio [aOR], 5.59; 95% confidence interval [CI], 1.64–19.02; *p* = 0.006) and a jaundice duration ≥ 14 days (aOR, 5.68; 95% CI, 1.61–20.04; *p* = 0.007) were independently associated with below-cutoff personal–social performance. **Conclusions:** Among term infants, NHB was associated with an increased risk of below-cutoff performance across several GDS-C domains, particularly those reflecting gross motor, personal–social and visual-spatial functions. Severe hyperbilirubinaemia and prolonged jaundice were independent risk factors. The GDS-C may serve as a sensitive, domain-specific instrument for the early identification of infants at risk of adverse neurodevelopmental outcomes.

## 1. Introduction

Neonatal hyperbilirubinaemia (NHB) affects approximately 60% of term and up to 80% of preterm infants during the first week of life and is one of the most common conditions encountered in neonatal care [1,2]. Although most cases are physiological and self-limiting, severe or prolonged hyperbilirubinaemia is a leading indication for neonatal hospital admission and may result in bilirubin-induced neurologic dysfunction (BIND)—a spectrum of injury ranging from acute bilirubin encephalopathy to kernicterus, characterised by choreoathetoid cerebral palsy, sensorineural hearing loss and gaze abnormalities [3,4,5]. Even in the absence of overt kernicterus, subclinical bilirubin neurotoxicity can give rise to subtle, long-term deficits in cognition, academic attainment and behaviour [6,7,8].

Unconjugated bilirubin exerts neurotoxicity through mitochondrial dysfunction, oxidative stress, excitotoxicity and impaired myelination; the basal ganglia, hippocampus and cerebellum are particularly vulnerable [3,8,9,10]. Of note, the regional selectivity of bilirubin injury may differentially affect specific developmental domains [4,8]. The threshold for neurotoxicity, however, remains poorly defined. Total serum bilirubin (TSB) concentrations alone are imperfect predictors of outcome, and the clinical signs of BIND are often subtle or absent during the neonatal period, making early identification challenging [3,11]. Although American Academy of Pediatrics (AAP) guidelines and the Bhutani nomogram have substantially reduced the incidence of kernicterus in high-income countries [2,12], long-term neurodevelopmental sequelae can persist even in infants who do not meet the criteria for exchange transfusion [4,13,14,15].

Accurate neurodevelopmental assessment tools are therefore essential to guide clinical follow-up. Although the Bayley Scales of Infant and Toddler Development are widely used in high-risk neonatal populations [16,17], the Griffiths Developmental Scales–Chinese Edition (GDS-C) provides a more comprehensive assessment across six distinct domains: Locomotor, Personal–Social, Hearing and Language, Eye and Hand Coordination, Performance and Practical Reasoning [18]. Each domain maps onto relatively distinct neuroanatomical substrates—the precentral gyrus and supplementary motor area for gross motor function, the frontal lobes for executive and social function, the language areas of the dominant hemisphere and fronto-striatal circuits for visual-spatial processing—thereby offering greater anatomical localising value when characterising bilirubin-related neurodevelopmental profiles [18]. Despite these advantages, evidence on the use of the GDS-C in infants with NHB remains scarce. We therefore undertook this retrospective cohort study to evaluate neurodevelopmental outcomes in term infants with NHB using the GDS-C, with three specific objectives: to compare developmental profiles between infants with NHB and healthy controls; to investigate the associations between the severity and duration of hyperbilirubinaemia and neurodevelopmental outcomes; and to identify independent risk factors for adverse outcomes.

## 2. Materials and Methods

### 2.1. Study Design and Participants

This single-centre, hospital-based retrospective cohort study was conducted at the Department of Paediatrics, Shanghai Sixth People’s Hospital (Lingang Campus)—a tertiary teaching hospital affiliated with Shanghai Jiao Tong University School of Medicine, located in the Lingang Special Area of the China (Shanghai) Pilot Free Trade Zone in southeastern Shanghai, China. The department serves as a regional referral centre for neonatal jaundice and a routine follow-up site for high-risk neonates from the Lingang Special Area and the surrounding districts of Pudong New Area. Eligible infants were identified consecutively from the hospital’s high-risk neonatal follow-up registry. Clinical records of full-term newborns delivered between September 2019 and August 2023 were reviewed, and neurodevelopmental assessments at follow-up were completed by 31 August 2024 (data lock). The study was approved by the Ethics Committee of Shanghai Sixth People’s Hospital (approval number: 2024-150-(01); dated 4 September 2024); the Institutional Review Board granted a waiver for retrospective record review, and prospective written informed consent was obtained from a parent or legal guardian of every child before the neurodevelopmental assessment.

**Figure 1:**
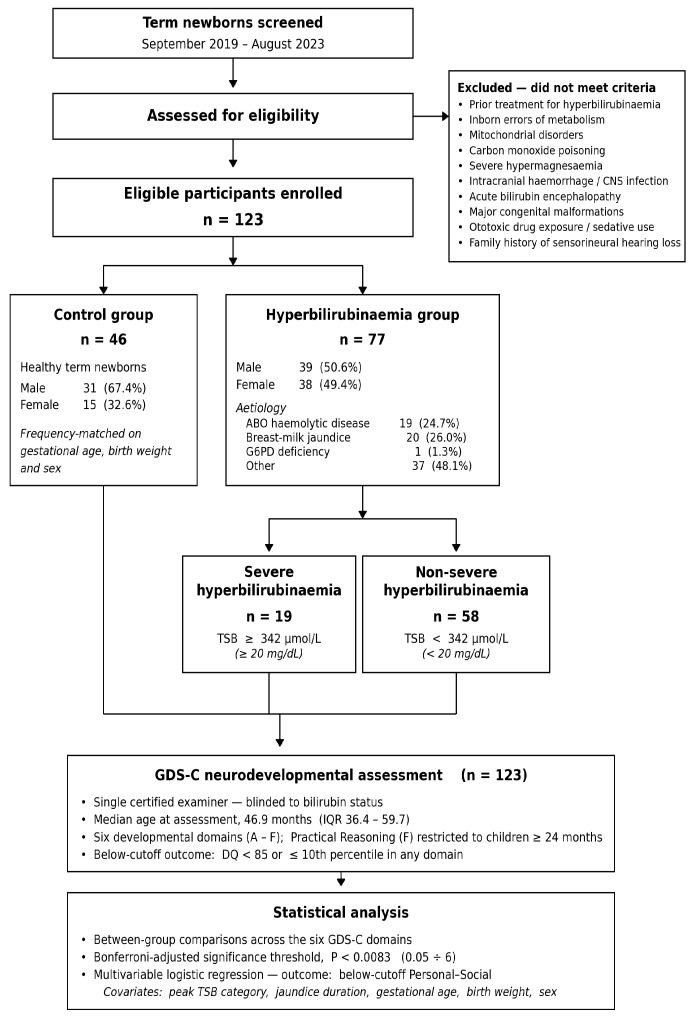
Study flow diagram. Retrospective cohort study conducted between September 2019 and August 2023. Medical records of full-term newborns admitted to the Sixth People’s Hospital, Shanghai Jiao Tong University School of Medicine, were reviewed. Infants were excluded for prior treatment before admission, inborn errors of metabolism, mitochondrial disorders, major congenital malformations, intracranial haemorrhage or central nervous system (CNS) infection, acute bilirubin encephalopathy, or ototoxic drug exposure. A total of 123 eligible participants were enrolled: 46 healthy controls (31 males, 15 females) and 77 infants with hyperbilirubinaemia (39 males, 38 females), frequency-matched by gestational age (37 + 0–41 + 6 weeks), birth weight and sex. The hyperbilirubinaemia group was further classified as severe (TSB ≥ 342 μmol/L [20 mg/dL]; *n* = 19) or non-severe (TSB < 342 μmol/L; *n* = 58). Aetiologies comprised ABO haemolytic disease (*n* = 19, 24.7%), breast milk jaundice (*n* = 20, 26.0%), G6PD deficiency (*n* = 1, 1.3%) and other causes (*n* = 37, 48.1%). All 123 infants underwent GDS-C neurodevelopmental assessment by a single certified examiner who was blinded to bilirubin status, at a median age of 46.9 months (IQR, 36.4–59.7). Statistical analyses included between-group comparisons with a Bonferroni-adjusted significance threshold of *p* < 0.0083 and multivariable logistic regression. GDS-C, Griffiths Developmental Scales–Chinese Edition; TSB, total serum bilirubin; CNS, central nervous system; G6PD, glucose-6-phosphate dehydrogenase.

Eligible infants met the following inclusion criteria: (1) gestational age between 37 + 0 and 41 + 6 weeks; (2) birth weight between 2500 g and 4000 g; (3) a diagnosis of NHB, defined as a TSB concentration exceeding the hour-specific phototherapy threshold of the AAP 2004 guidelines [2], a rise in TSB of more than 85 μmol/L per day or 8.5 μmol/L per hour, or jaundice persisting for more than two weeks with a predominantly unconjugated pattern; (4) no evidence of intracranial injury secondary to haemorrhage or central nervous system infection; (5) no major congenital malformations; (6) no history of ototoxic drug exposure, sedative use or family history of sensorineural hearing loss; and (7) no clinical features of acute bilirubin encephalopathy at presentation or during hospitalisation. Exclusion criteria were prior treatment for hyperbilirubinaemia, confirmed or suspected inborn errors of metabolism, mitochondrial disorders, carbon monoxide poisoning or severe hypermagnesaemia. A participant flow diagram is presented in Figure 1.

The control group comprised 46 healthy full-term newborns (31 males, 15 females) with neither clinical nor biochemical evidence of jaundice and was frequency-matched to the jaundice group by gestational age (within one week), birth weight (within 300 g) and sex.

### 2.2. Neurodevelopmental Assessment

Neurodevelopmental outcomes were assessed using the GDS-C (Hogrefe Ltd., Oxford, UK), a standardised, norm-referenced instrument that evaluates six developmental domains: Locomotor (gross motor), Personal–Social, Hearing and Language, Eye and Hand Coordination (fine motor), Performance (visual-spatial processing and executive function) and Practical Reasoning [18,19]. In children younger than two years, five domains (A–E) are assessed; the Practical Reasoning domain is added for children aged two years and older. All assessments were performed by a single certified GDS-C examiner who was blinded to the participant’s bilirubin status. Testing began with an entry item approximately two months below the child’s chronological age; the examiner then proceeded upwards or downwards until six consecutive passes (ceiling) or six consecutive failures (floor) were recorded.

For each domain, a developmental quotient (DQ) was calculated according to the GDS-C normative data. A DQ below 85 (more than one standard deviation below the mean of 100) or a score at or below the 10th percentile was classified as ‘below cutoff’ and regarded as indicative of developmental delay. The primary outcome was the proportion of infants scoring below cutoff in one or more domains.

### 2.3. Definitions and Statistical Analysis

Severe hyperbilirubinaemia was defined as a TSB ≥ 342 μmol/L (20 mg/dL), in accordance with the threshold recommended by AAP 2004 guidelines [2]. Jaundice duration was categorised as short (<7 days), moderate (7–14 days) or prolonged (≥14 days).

Data were analysed using IBM SPSS Statistics version 22.0 (IBM Corp., Armonk, NY, USA). All statistical tests were two-sided. Because six domains were tested simultaneously, a Bonferroni correction was applied, yielding an adjusted significance threshold of *p* < 0.0083 (0.05 ÷ 6). Normally distributed continuous variables are presented as means (standard deviation) and were compared using the independent-samples *t*-test; non-normally distributed continuous variables are presented as medians (interquartile range [IQR]) and were compared using the Mann–Whitney U test. Categorical variables are presented as numbers (percentage) and were compared using the chi-squared test with Yates’ continuity correction when the minimum expected cell count was ≥5 and with Fisher’s exact test when the minimum expected count was <5. Bivariate associations between peak TSB and domain-specific DQ scores were assessed using Pearson correlation coefficients.The Personal–Social domain was pre-specified as the single primary dependent variable for multivariable analysis on the basis of two considerations: (i) a priori biological plausibility—the Personal–Social subscale evaluates social adaptation and emergent executive function, behaviours that depend on the integrity of frontal–subcortical circuits (particularly the basal ganglia, thalamus and their prefrontal projections), which are classical anatomical targets of bilirubin neurotoxicity [3,8,10]; and (ii) within-cohort univariate findings—across the jaundice-versus-control, severity-graded and jaundice-duration comparisons, the Personal–Social domain showed the most consistent and graded association with bilirubin exposure, and was the only domain whose unadjusted severity comparison (*p* = 0.003) approached the Bonferroni-corrected significance threshold (adjusted *p* = 0.018). Restricting the multivariable analysis to a single pre-specified primary outcome was intended to minimise the risk of post hoc selection bias and the type I error inflation that would arise from fitting six parallel domain-specific models. Multivariable logistic regression was therefore performed with below-cutoff performance on the Personal–Social domain as the dependent variable. Covariates included peak TSB category (≥342 vs. <342 μmol/L), jaundice duration (≥14 vs. <14 days), gestational age, birth weight and infant sex. Adjusted odds ratios (aOR) with 95% confidence intervals (CI) are reported.

## 3. Results

### 3.1. Participant Characteristics

Between September 2019 and August 2023, 123 full-term newborns were enrolled: 77 (62.6%) with NHB (jaundice group: 39 males, 38 females) and 46 (37.4%) healthy controls (31 males, 15 females). In the jaundice group, the aetiological diagnoses included ABO haemolytic disease (*n* = 19, 24.7%), breast milk jaundice (*n* = 20, 26.0%), glucose-6-phosphate dehydrogenase (G6PD) deficiency (*n* = 1, 1.3%) and other causes (*n* = 37, 48.1%). The overall male-to-female ratio was 1.3:1 (70 males, 53 females). No statistically significant between-group differences were observed for gestational age, birth weight or age at assessment (all *p* > 0.05). Neurodevelopmental assessment was performed at a median age of 46.9 months (IQR, 36.4–59.7) (Table 1).

### 3.2. GDS-C Domain Comparisons: Jaundice Versus Control Group

Compared to the control group, the jaundice group had higher proportions of below-cutoff performance in four of the six domains: Locomotor (26.0% vs. 10.9%; *p* = 0.045), Personal–Social (28.6% vs. 10.9%; *p* = 0.022), Eye and Hand Coordination (29.9% vs. 10.9%; *p* = 0.015) and Performance (35.1% vs. 15.2%; *p* = 0.018). After Bonferroni correction, the differences in the Hearing and Language (31.2% vs. 26.1%; *p* = 0.551) and Practical Reasoning (7.8% vs. 17.4%; *p* = 0.121) domains did not reach statistical significance (Table 2).

### 3.3. Severe Hyperbilirubinaemia Subgroup Analysis

Within the jaundice group, infants with severe hyperbilirubinaemia (TSB ≥ 342 μmol/L, *n* = 19) were compared to those with non-severe hyperbilirubinaemia (TSB < 342 μmol/L, *n* = 58). The severe subgroup had higher proportions of below-cutoff performance in the Locomotor (47.4% vs. 19.0%; *p* = 0.032), Personal–Social (57.9% vs. 19.0%; *p* = 0.003) and Performance (57.9% vs. 27.6%; *p* = 0.034) domains, although only the Personal–Social domain comparison approached the Bonferroni-adjusted significance threshold (adjusted *p* = 0.018). Differences in the Hearing and Language (42.1% vs. 27.6%; *p* = 0.368), Eye and Hand Coordination (47.4% vs. 24.1%; *p* = 0.103) and Practical Reasoning (50.0% vs. 16.7%; *p* = 0.139) domains did not reach statistical significance (Table 3).

### 3.4. Jaundice Duration and Phototherapy Timing

A statistically significant association was observed between jaundice duration and personal–social outcomes (*p* = 0.007), with prolonged jaundice (≥14 days) associated with poorer performance. The interval from jaundice onset to initiation of phototherapy was also associated with personal–social outcomes (p = 0.010). Subgroup analyses according to the timing of jaundice onset (early, <24 h, *n* = 22 vs. late, ≥24 h, *n* = 55) and the timing of phototherapy initiation (early, ≤1 day, *n* = 28; intermediate, 2 days, *n* = 19; delayed, ≥3 days, *n* = 30) revealed no statistically significant differences across domains (all *p* > 0.05) (Table 4).

### 3.5. Multivariable Regression Analysis

Multivariable logistic regression was performed with below-cutoff personal–social performance as the dependent variable, with adjustment for gestational age, birth weight, infant sex, peak TSB category and jaundice duration. A peak TSB ≥ 342 μmol/L (aOR, 5.59; 95% CI, 1.64–19.02; *p* = 0.006) and a jaundice duration ≥ 14 days (aOR, 5.68; 95% CI, 1.61–20.04; *p* = 0.007) were independently associated with below-cutoff personal–social performance (Table 5).

## 4. Discussion

In this retrospective cohort study, the GDS-C was used to evaluate neurodevelopmental outcomes in term infants with NHB. Our principal findings were that infants with a history of hyperbilirubinaemia had higher proportions of below-cutoff performance than controls in the Locomotor, Personal–Social, Eye and Hand Coordination and Performance domains; that severe hyperbilirubinaemia (TSB ≥ 342 μmol/L) was independently associated with below-cutoff performance in the Locomotor, Personal–Social and Performance domains; and that prolonged jaundice (≥14 days) was an independent risk factor for below-cutoff personal–social performance. These findings are consistent with the known regional selectivity of bilirubin neurotoxicity and underscore the importance of structured developmental surveillance in this high-risk population [3,4,8].

The observed pattern of domain-specific vulnerability is biologically plausible. The basal ganglia, hippocampus and cerebellum—regions that are critical for motor coordination, procedural learning and executive function—are particularly susceptible to bilirubin-induced injury because of their high metabolic demand and selective neuronal vulnerability [3,8,9]. Below-cutoff performance in the Locomotor domain probably reflects injury to the basal ganglia and the cerebellar pathways that support gross motor coordination [8]. Deficits in the Performance domain, which assesses visual-spatial processing and working memory, may indicate dysfunction of fronto-striatal and cerebello-cortical circuits [3]. The Personal–Social domain, which evaluates social adaptation and executive function, showed the strongest association with both the severity and duration of hyperbilirubinaemia. This may reflect the vulnerability of subcortical structures, including the basal ganglia and the thalamus, which are important for social cognition and behavioural regulation during early development [4,8]. The almost six-fold increase in the odds of below-cutoff personal–social performance associated with severe hyperbilirubinaemia (adjusted OR 5.59) suggests that this domain may be particularly sensitive to clinically meaningful bilirubin exposure. Prolonged jaundice was similarly associated with below-cutoff personal–social performance (adjusted OR 5.68), a finding that may reflect sustained bilirubin exposure during a critical period of maturation of fronto-subcortical circuits [8,20].

Conversely, no statistically significant between-group differences were observed in the Hearing and Language and Practical Reasoning domains. The absence of statistical significance, however, cannot be interpreted as evidence of no effect. Our modest sample size limited the statistical power to detect small-to-moderate effects, and the possibility of type II error must be acknowledged. Injury to the auditory pathway—particularly the brainstem auditory nuclei—is a well-documented consequence of severe hyperbilirubinaemia, and sensorineural hearing loss is a hallmark feature of kernicterus [3,21]. The lack of significant findings in our cohort may therefore reflect the relative rarity of overt hearing impairment in infants with moderate hyperbilirubinaemia who do not develop severe BIND, or the possibility that language development at this age is substantially modulated by environmental factors [22]. The Practical Reasoning subscale may have been limited by the young age at assessment, at which abstract reasoning skills are only beginning to emerge, which may have constrained sensitivity to subtle impairment [18]. Alternatively, the cortical association areas assessed using the Practical Reasoning subscale may be relatively spared by bilirubin at moderate exposure levels [3,8]. Longitudinal follow-up into the school years may therefore be required to determine whether delayed effects on higher-order cognitive functions become apparent as age and cognitive demands increase [8,20,23].

Our findings are broadly consistent with previous reports of an association between neonatal hyperbilirubinaemia and adverse neurodevelopmental outcomes. Le Pichon and colleagues described a correlation between peak TSB concentrations and neurobehavioural abnormalities, suggesting a dose–response relationship between bilirubin exposure and acute neurological dysfunction [24]. El-Tatawy and colleagues reported that severe hyperbilirubinaemia was associated with lower intelligence quotient scores, with more severe hyperbilirubinaemia corresponding to greater cognitive impairment [6]. Our results extend these observations by providing a granular, domain-specific characterisation of developmental vulnerability using the GDS-C. Some discrepancies, however, deserve comment. Earlier studies reported significant impairments in Hearing and Language and Eye and Hand Coordination [6,25] that did not reach statistical significance in our cohort, which may reflect differences in study design, the distribution of disease severity or the choice of assessment instrument [6,18]. The GDS-C comprises six subscales, each mapping onto distinct neuroanatomical substrates, which may confer greater localising value than instruments that assess fewer domains [18,19].

Several clinical implications follow from these findings. First, term infants with a history of clinically significant hyperbilirubinaemia should be regarded as a high-risk population who warrant structured developmental surveillance during early childhood, even in the absence of acute bilirubin encephalopathy. Second, a peak TSB ≥ 342 μmol/L and jaundice persisting ≥14 days are clinically accessible risk markers that can be used to prioritise infants for comprehensive developmental assessment and early intervention. Third, the domain-specific pattern of impairment—particularly the involvement of motor, visual-spatial and social-adaptive functions—indicates that follow-up assessments should be broad-based rather than focused exclusively on cognition or language.

Several limitations should be acknowledged. First, the single-centre, retrospective design may introduce selection bias and limits generalisability. The modest sample size (*n* = 123) further constrained statistical power, particularly for subgroup analyses, so that non-significant domain differences should be interpreted with caution rather than taken as definitive evidence of no effect. Second, as a retrospective study based on medical record review, our data are subject to information bias, including incomplete documentation and variability in clinical recording practice. We were unable to systematically collect data on potentially important confounders, including parental socioeconomic status, maternal education, home environment quality and feeding practices, all of which can substantially influence neurodevelopmental outcomes [8,20]. Third, classifying developmental outcomes by binary cutoffs rather than by continuous standardised scores reduces granularity and statistical efficiency. Fourth, the heterogeneity of aetiologies within the jaundice group—encompassing ABO haemolytic disease, breast milk jaundice, G6PD deficiency and other causes—represents a source of clinical and biological variability that we were unable to address adequately, and the neurodevelopmental risks associated with these aetiologies may differ considerably owing to variation in bilirubin kinetics and associated comorbidities [23]. Fifth, neurodevelopmental assessment was performed at a single time point and therefore provides only a cross-sectional snapshot; longitudinal follow-up is required to clarify whether the observed developmental differences persist, evolve or resolve [8,20]. Replication in larger, multicentre, prospectively designed cohorts with comprehensive ascertainment of covariates will be needed to confirm and extend these findings.

In this retrospective cohort study of 123 term infants, neonatal hyperbilirubinaemia was associated with higher proportions of below-cutoff performance in the Locomotor, Personal–Social, Eye and Hand Coordination and Performance domains of the GDS-C. Severe hyperbilirubinaemia (TSB ≥ 342 μmol/L) and prolonged jaundice (≥14 days) were independent risk factors for below-cutoff personal–social performance. These findings support the use of the GDS-C as a sensitive, domain-specific instrument for developmental surveillance in infants with neonatal hyperbilirubinaemia and underscore the need for structured follow-up programmes targeting infants with severe or prolonged jaundice.

## Figures and Tables

**Table 1:** Baseline demographic and clinical characteristics of the study population.

Characteristic	Jaundice Group (*n* = 77)	Control Group (*n* = 46)	*p*-Value
Demographic characteristics			
Male sex—no. (%)	39 (50.6)	31 (67.4)	0.060
Gestational age—weeks *	37^+0^–41^+6^	37^+0^–41^+6^	NS
Birth weight—g *	2500–4000	2500–4000	NS
Age at neurodevelopmental assessment—mo, median (IQR)	46.9 (36.4–59.7)	46.9 (36.4–59.7)	NS
Aetiology of hyperbilirubinaemia—no. (%)			
ABO haemolytic disease	19 (24.7)	—	—
Breast milk jaundice	20 (26.0)	—	—
G6PD deficiency	1 (1.3)	—	—
Other or undetermined	37 (48.1)	—	—
Severity of hyperbilirubinaemia—no. (%)			
Severe (TSB ≥ 342 μmol/L)	19 (24.7)	—	—
Non-severe (TSB < 342 μmol/L)	58 (75.3)	—	—

Continuous variables are presented as medians (interquartile range, IQR); categorical variables are presented as numbers (percentage). *p*-Values were derived from the chi-squared test or Fisher’s exact test for categorical variables and the Mann–Whitney U test for continuous variables. * Values are presented as ranges, as defined by the inclusion criteria; gestational age is expressed as completed weeks + days (e.g., 37^+0^ denotes 37 weeks and 0 days). G6PD, glucose-6-phosphate dehydrogenase; IQR, interquartile range; NS, not significant (*p* > 0.05); TSB, total serum bilirubin.

**Table 2:** Proportion of infants scoring below the developmental cutoff in each GDS-C domain: jaundice group versus controls.

GDS-C Domain	Jaundice Group (*n* = 77)—No. (%)	Control Group (*n* = 46)—No. (%)	*p*-Value	Adjusted *p*-Value *
Locomotor (A)	20 (26.0)	5 (10.9)	0.045	0.270
Personal–Social (B)	22 (28.6)	5 (10.9)	0.022	0.132
Hearing and Language (C)	24 (31.2)	12 (26.1)	0.551	1.000
Eye and Hand Coordination (D)	23 (29.9)	5 (10.9)	0.015	0.090
Performance (E)	27 (35.1)	7 (15.2)	0.018	0.108
Practical Reasoning (F) †	6 (7.8)	2 (4.3)	0.121	0.726

Values represent the number (and percentage) of infants scoring below the GDS-C developmental cutoff (developmental quotient < 85 or ≤10th percentile). *p*-Values were derived from the chi-squared test or Fisher’s exact test, as appropriate. * Bonferroni-adjusted *p*-values are reported to account for the simultaneous testing of six developmental domains; the adjusted significance threshold was *p* < 0.0083. † The Practical Reasoning subscale is administered only to children aged ≥24 months; the denominators for this row were therefore restricted to children who had reached this age (jaundice group, *n* = 24; control group, *n* = 10). Letters in parentheses (A–F) denote the six subscale codes of the Griffiths Development Scales as originally designated. GDS-C, Griffiths Development Scales–Chinese edition.

**Table 3:** Proportion of infants scoring below the developmental cutoff in each GDS-C domain, stratified by the severity of hyperbilirubinaemia.

GDS-C Domain	Severe (TSB ≥ 342 μmol/L, *n* = 19)—No. (%)	Non-Severe (TSB < 342 μmol/L, *n* = 58)—No. (%)	*p*-Value	Adjusted *p*-Value *
Locomotor (A)	9 (47.4)	11 (19.0)	0.032	0.190
Personal–Social (B)	11 (57.9)	11 (19.0)	0.003	0.018
Hearing and Language (C)	8 (42.1)	16 (27.6)	0.368	1.000
Eye and Hand Coordination (D)	9 (47.4)	14 (24.1)	0.103	0.617
Performance (E)	11 (57.9)	16 (27.6)	0.034	0.201
Practical Reasoning (F) †	3 (50.0)	3 (16.7)	0.139	0.835

Values represent the number (and percentage) of infants scoring below the GDS-C developmental cutoff (developmental quotient < 85 or ≤10th percentile). *p*-Values were derived from Fisher’s exact test when the minimum expected cell count was <5 and otherwise from the chi-squared test with Yates’ continuity correction. * Bonferroni-adjusted *p*-values are reported to account for the simultaneous testing of six developmental domains; the adjusted significance threshold was *p* < 0.0083. † The Practical Reasoning subscale was administered only to children aged ≥24 months; the denominators for this row were therefore restricted to children who had reached this age (severe subgroup, *n* = 6; non-severe subgroup, *n* = 18); percentages are calculated using these restricted denominators. Letters in parentheses (A–F) denote the six subscale codes of the Griffiths Development Scales as originally designated. GDS-C, Griffiths Development Scales–Chinese edition; TSB, total serum bilirubin.

**Table 4:** Subgroup analyses of the effects of the timing of jaundice onset, the timing of phototherapy initiation and the duration of jaundice on GDS-C domain outcomes.

Subgroup Comparison	Locomotor (A)	Personal–Social (B)	Hearing and Language (C)	Eye and Hand Coordination (D)	Performance (E)	Practical Reasoning (F)
Timing of jaundice onset						
<24 h (*n* = 22) vs. ≥24 h (*n* = 55)	0.327	0.205	0.643	0.051	0.708	0.078
Timing of phototherapy initiation						
≤1 day (*n* = 28) vs. 2 days (*n* = 19) vs. ≥3 days (*n* = 30)	0.802	0.352	0.467	0.832	0.591	0.475
Duration of jaundice						
<7 days (*n* = 38) vs. 7–14 days (*n* = 33) vs. ≥14 days (*n* = 6)	0.467	0.007 *	0.091	0.084	0.070	0.251
Interval from jaundice onset to phototherapy						
≤1 day (*n* = 28) vs. 2 days (*n* = 19) vs. ≥3 days (*n* = 30)	0.212	0.010	0.701	0.074	0.072	0.134

All cell values are *p*-values. *p*-Values were derived from the Mann–Whitney U test for two-group comparisons and the Kruskal–Wallis H test for three-group comparisons. * Reaches statistical significance after Bonferroni adjustment for the simultaneous testing of six developmental domains (adjusted threshold, *p* < 0.0083). Letters in parentheses (A–F) denote the six subscale codes of the Griffiths Development Scales as originally designated. GDS-C, Griffiths Development Scales–Chinese edition.

**Table 5:** Multivariable logistic regression model of independent predictors of below-cutoff performance on the Personal–Social domain of the GDS-C.

Variable	Reference Category	Adjusted OR (95% CI)	*p*-Value
Primary Exposures of Interest			
Severe hyperbilirubinaemia (TSB ≥ 342 μmol/L)	TSB < 342 μmol/L	5.59 (1.64–19.02)	0.006
Prolonged jaundice (≥14 days)	<14 days	5.68 (1.61–20.04)	0.007

The dependent variable was below-cutoff performance for the Personal–Social domain of the GDS-C (developmental quotient < 85 or ≤10th percentile). The multivariable logistic regression model was adjusted for gestational age (per week), birth weight (per 100 g) and infant sex (male vs. female). Because the cohort was frequency-matched on these three variables by design, the adjusted effect estimates of the covariates approached unity and none reached statistical significance (all *p* > 0.10); for clarity, only the primary exposures of interest are reported in the table. aOR, adjusted odds ratio; CI, confidence interval; GDS-C, Griffiths Development Scales–Chinese edition; TSB, total serum bilirubin.

## Data Availability

The datasets generated and/or analysed during the present study are not publicly available due to ethical restrictions and institutional policy aimed at protecting patient privacy. De-identified data may be obtained from the corresponding author on reasonable request, subject to approval by the Ethics Committee of the Shanghai Sixth People’s Hospital. Researchers wishing to access the data should submit a formal request outlining the purpose and scope of the proposed analysis; requests will be evaluated in accordance with the ethical guidelines governing the present study.
